# Biobehavior Life Regulation (BLR) scale for living well in chronic pain: Preliminary scale development and validation

**DOI:** 10.1371/journal.pone.0299126

**Published:** 2024-04-29

**Authors:** Aram S. Mardian, Martha Kent, Jenna L. Gress-Smith, Lucia Ciciolla, Morgan L. Regalado-Hustead, Brandon A. Scott, Megan E. Petrov

**Affiliations:** 1 Chronic Pain Wellness Center, Phoenix VA Health Care System, Phoenix, AZ, United States of America; 2 Department of Family, Community and Preventive Medicine, University of Arizona College of Medicine–Phoenix, Phoenix, AZ, United States of America; 3 Research Department, Phoenix VA Health Care System, Phoenix, AZ, United States of America; 4 Department of Psychology, Arizona State University, Tempe, AZ, United States of America; 5 Department of Psychology, Phoenix VA Health Care System, Phoenix, AZ, United States of America; 6 Department of Psychology, Oklahoma State University, Stillwater, OK, United States of America; 7 Department of Educational Psychology, Northern Arizona University, Flagstaff, AZ, United States of America; 8 Edson College of Nursing and Health Innovation, Arizona State University, Phoenix, AZ, United States of America; University of Pecs Medical School, HUNGARY

## Abstract

Currently available pain assessment scales focus on pain-related symptoms and limitations imposed by pain. Validated assessment tools that measure how pain is regulated by those who live well with pain are missing. This study seeks to fill this gap by describing the development and preliminary validation of the Biobehavior Life Regulation (BLR) scale. The BLR scale assesses engagement, social relatedness, and self-growth in the presence of chronic pain and the unpredictability of chronic pain. Sources for items included survivor strategies, patient experiences, existing scales, and unpredictable pain research. Review for suitability yielded 52 items. Validation measures were identified for engagement, social relatedness, self-growth, and unpredictability of pain. The study sample (n = 202) represented patients treated in the Phoenix VA Health Care System (n = 112) and two community clinics (n = 90). Demographic characteristics included average age of 52.5, heterogeneous in ethnicity and race at the VA, mainly Non-Hispanic White at the community clinics, 14 years of education, and pain duration of 18 years for the VA and 15.4 years for community clinics. Exploratory factor analysis using Oblimin rotation in the VA sample (n = 112) yielded a two-factor solution that accounted for 48.23% of the total variance. Confirmatory factor analysis (CFA) in the same sample showed high correlations among items in Factor 1, indicating redundancy and the need to further reduce items. The final CFA indicated a 2-factor solution with adequate fit to the data. The 2-factor CFA was replicated in Sample 2 from the community clinics (*n* = 90) with similarly adequate fit to the data. Factor 1, Pain Regulation, covered 8 items of engagement, social relatedness, and self-growth while Factor 2, Pain Unpredictability, covered 6 items related to the experience of unpredictable pain. Construct validity showed moderate to higher Pearson correlations between BLR subscales and relevant well-established constructs that were consistent across VA and community samples. The BLR scale assesses adaptive regulation strategies in unpredictable pain as a potential tool for evaluating regulation resources and pain unpredictability.

## Introduction

Pain is an essential function of homeostatic regulation that signals danger to the organism [1–6]. However, currently available pain assessment scales have focused mainly on the perceived effects of symptoms of pain based on models of pain care that focus on symptom reduction, as exemplified by the Initiative on Methods, Measurement, and Pain Assessment in Clinical Trials (IMMPACT) [7]. The concept of homeostatic regulation has not occupied a prominent place in pain research. Thus it is not surprising that pain as a part of the broad life regulation process is missing from the pain assessment literature. The detailed work of Craig identified pain as a regulatory “homeostatic emotion” [1, 2, 6]. With the development of the Biobehavior Life Regulation (BLR) scale for chronic pain, we seek to place pain back into the context of homeostatic life regulation. This also places homeostasis into a larger complex systems perspective with a control model of feedback and feedforward control [8]. Homeostasis is a feedback, closed-loop process that regulates set points much like a thermostat does. It is reactive to pain stimuli feedback. The BLR scale aims to measure, in a graded and scaled manner, the feedforward anticipatory control that allows participants to identify the regulatory resources they perceive to have despite pain.

The neural network that may be particularly supportive of life regulation is the default network (DN). The DN is activated by self-guided thought that is not focused on the execution of a task but is decoupled from attention-demanding activities and from salient stimuli such as pain. The decoupling allows self-generated thinking to explore ways of being well despite pain. An extensive literature shows positive correlations of DN with such scales as curiosity [9], compassion [10] or creativity [11]. The DN’s capacity for simulation and imagining alternatives or the future point to the DN as a potential life regulation cortical network.

As a tool, the BLR scale can assess adaptive regulation in the context of pain and help to evaluate what living well in a pain environment might be. The focus of this study is the development of a scale to assess the regulatory resources that are engaged for living well in pain and help clarify the adaptive regulatory features for optimal functioning in the experience of chronic pain.

### Models, clinical treatment, and assessment scales for pain

George Box’s quote “all models are wrong but some are useful” [12] is particularly pertinent to chronic pain. Ideally, a coherent and useful model of pain informs both clinical treatments and assessment scales used to better understand an individual’s experience of chronic pain and response to treatment. Debate about the nature of pain has been longstanding and current clinical treatments and available assessment scales are based on different models of pain. A mechanical, biomedical model of pain has largely dominated the clinical treatment and assessment of pain since the Enlightenment and advent of modern medicine. In this model, pain is seen as a symptom that represents a passive measure of tissue damage in the body. Medications, invasive procedures, and surgeries aimed at reducing symptoms of pain by disrupting what are felt to be the specific aspects of the nervous system that produce pain are the primary clinical treatments of this model; and pain intensity, measured by the Numeric Rating Scale, is the quintessential assessment measure for the biomedical model [13]. Models that expand beyond nociception include the gate control and neuromatrix theories of pain [14, 15] and the biopsychosocial [16] and sociopsychobiological [17, 18] models. While these models incorporate a broader array of factors that influence the experience of pain, treatment approaches have often continued to aim at symptom reduction as in the operant conditioning methods of Fordyce [19], the fear avoidance model of Vlaeyen and Linton [20], the motivational goal-directed approach pioneered by Karoly and Ruehlman [21], and Cognitive Behavioral Therapy. Sturgeon and Zautra [22] championed resilience as the “new paradigm” for the study of pain and considered “three primary classes of resilient outcomes: recovery, sustainability, and growth.” New therapeutic approaches directed the focus away from pain to adaptive tools and resources including mindfulness [23], finding benefits [24] and Acceptance and Commitment Therapy (ACT) [25]. These changed the focus from symptom reduction to capacities that were independent of pain. No longer could pain be seen only through the lens of symptoms and limitations. We join the continued debate about the nature of pain and how to treat and assess it. We add our own clinical biobehavioral life regulation intervention [26], and identify the need for assessing life regulation and unpredictability of pain with the proposed Biobehavior Life Regulation (BLR) scale for pain. The BLR approach to theory, intervention, and assessment shifts the focus from disease, symptoms, and limitations to enhancing a person’s inner adaptive regulatory capacities that are anticipatory rather than reactive within the context of salient, aversive, unpredictable environments. The purpose of the BLR scale is to assess adaptive regulation in the face of aversive unpredictable pain.

### Chronic pain as aversive and unpredictable

Chronic pain has long been recognized as inherently unpredictable and ambiguous [27]. We view chronic pain as a perceived internal aversive environment that shares basic features with external threatening environments, notably aversive unpredictability and survival threat. An extensive experimental literature has long investigated the effects of unpredictable pain in rodents [28]. In humans, long established findings demonstrate that unpredictability is associated with more anxiety and fear [29–31], lower quality of life [32], greater pain [33], anticipatory pain-related fear [34–36], response biases that maintain pain and fear [37], and generalizes to other contexts [38]. The unpredictable aversive features of chronic pain are also characteristic of extreme human-made environments of prisoners of war (POW), the holocaust, and the Gulag. This recognition guided us to view pain as a body environment that the person lives in and to consider adaptive responses to persistent aversive unpredictability.

### Adaptation in aversive unpredictable environments

Our approach to chronic pain partly resembles Eccleston’s call to understand pain from the non-pathological perspective of how a “normal” person responds to pain [39], However, “normal” adaptive responses to chronic pain are not described by Eccleston and have not been described in the chronic pain literature. An approximation to “normal” responses could be found in those situations that are aversive and unpredictable, particularly the insiders’ subjective experience of such situations. We do have access to survivors’ subjective experiences as they describe their lives as insiders and not as responders to interviews or questionnaires. The insider observer makes visible what is invisible to the outside observer. In our early reviews of adaptive survival, we recognized survivors’ strategies as resembling biological regulation. Studies of biological regulation identified the purpose of regulation to be the preservation of life [40, 41]. Regulation was particularly engaged by perturbations and threats from the environment, hence the need for regulation to preserve the life of the organism. The survivors’ adaptive efforts were their best attempts to stay alive. Thus we propose biobehavior life regulation as the mechanism doing the regulating in the aversive unpredictable internal body environment of chronic pain.

### Existing pain scales

The above described diversity of pain care models has resulted in the development of a large heterogeneous body of pain scales and a need for consensus that was addressed by IMMPACT [7]. Pain scales vary widely along a number of dimensions: (1) single domain to multidimensionality, (2) pain specificity and generality, (3) pain risk and prevention factors, and (4) disease specific pain measures. Multidimensional pain scales aim to comprehensively assess the experience of pain and response to treatment.

### Multidimensional pain scales

Three widely used multidimensional pain scales include: The West Haven-Yale Multidimensional Pain Inventory (WHYMPI/MPI) [42], the Pain Outcomes Questionnaire-VA (POQ-VA) [43], and the Profile of Chronic Pain: Extended Assessment Battery (PCP:EA) [44]. The WHYMPI is comprised of 52 items that cover five dimensions: perceived interference by pain in life roles, support from significant other, pain severity, perceived life control, and affective distress. These subscales assess the limitations that pain imposes. The POQ-VA and its 20 items covers six subscales: pain intensity, mobility, activities of daily living, vitality, negative affect, and fear. The content of POQ-VA and WHYMPI overlap in covering limitations caused by pain. The PCP:EA is a newer measure that generates a profile of chronic pain. Its 13 short subscales again assess limitations associated with pain, and also four items assess adaptive ignoring of pain, task persistence, and positive self-talk. Of note, these multidimensional scales focus on assessing an individual’s reactions to pain.

### New directions in pain assessment

Our BLR approach to chronic pain has some similarities with the mindful acceptance of the present moment and behavioral activation aspects of ACT. An ACT approach to chronic pain promotes willingness to experience aversive internal sensations, thoughts and emotions while an individual takes actions towards one’s values [25]. The eight item Chronic Pain Acceptance Questionnaire (CPAQ-8) represents a significant departure from the symptom and deficit focused scales.

While our BLR approach and ACT both seek to promote sustained positive activities in the presence of aversive internal experiences such as chronic pain, the BLR approach differs in a number of important ways. First, ACT focuses on global processes of acceptance and behavioral activation; it does not identify or train more specific processes of living well. BLR, on the other hand, is primarily focused on identifying and training the core life processes responsible for adaptive thriving–specifically the domains of engagement (exemplified by interest, curiosity, appreciation, and noticing beauty), social relatedness (exemplified by empathy, compassion, helping, friendship, and love), and self growth. Second, whereas the unifying feature of ACT is psychological flexibility in which an individual seeks to be open to both positive and negative experiences and takes valued actions independent of these experiences, BLR aims to train individuals how to use their innate life regulation capacities to live well within aversive unpredictable environments. The third and most striking difference is that BLR is based on a control systems model that assesses the extent of anticipatory orientation to pain through engagement, social relatedness, and self-growth that reach beyond pain for information, social connection, and thriving beyond the confines of pain. These are gradient in nature, open-looped, and do not specify goals or set points. The BLR scale aims to show the type and quality of anticipatory resources used by an individual within the context of chronic pain as well as the extent of the aversive unpredictability of the body environment. ACT focuses on the individual’s values and committed action towards those values and the CPAQ-8 does not include domains measuring specific anticipatory resources used by individuals or for the aversive unpredictability of the pain body environment. These differences suggest a continued gap in pain assessments for identifying and measuring adaptive life regulation processes within the context of aversive internal and external environments.

### The biobehavior life regulation scale

No existing pain assessment tools aim to identify and measure the adaptive strategies for living well inside their pain body world. The goal of the BLR scale is to fill this gap by capturing positive adaptation as well as the unpredictability of pain in the subjective experiences of chronic pain. The BLR scale assesses persons’ subjective strategies for living inside their pain world. These include the adaptive regulation resources available to individuals with chronic pain and the extent of unpredictability of chronic pain. This assessment of resources could contribute to an understanding of the adaptive features, behaviors, and thoughts of persons in chronic pain environments and help identify capacities that keep people well.

## Methods

### Qualitative thematic development

#### Adaptive survival in extreme environments

The development of the BLR scale draws on twenty years of our work that included examining adaptive survival in extreme human-made environments as described in the classical autobiographical texts of POW, holocaust, and Gulag survivors. Our qualitative approach to these texts informed the development of an intervention that trained adaptive responding to PTSD [45] and chronic pain [26]. The same qualitative approach informed the development of the BLR scale. The most important information that guided our qualitative approach came from the written texts where, on rare occasion, the writers described, however briefly, what helped them endure. This allowed us to let the themes emerge from the actions of survivors in a grounded theory approach using an inductive method. Two sets of expert survivor sources informed our thematic analysis:

1. Classic autobiographical texts of the holocaust by: Primo Levi [46], Charlotte Delbo [47], and Victor Frankl [48]; Texts of the Gulag by Eugenia Ginzburg [49] and Aleksandr Solzhenitsyn [50]. American sources included a televised documentary about American former POWs featuring Robert Shumaker [51] and Jim Stockdale’s text about POW experiences in Hanoi [52].
2. Survivor witnesses and family members who are known to survivor families of the holocaust by: (a) Cynthia Stonnington, and her Steininger family, as documented in letters by Jacques and Auguste Steininger [53], a diary by Heinz Steininger [54], and a family history by Susan Soyinka [55]; and (b) of co-author Martha Kent as documented in letters by Wanda Young [56] of post-war Polish/Soviet concentration camp and letters by Jasi Kordowskiei [57] and Aurelia Rymarkiewicz [58]; an autobiography by Martha Kent [59] and an ethnic cleansing paper [60].

Table 1 lists seven classical texts where survivors specify those approaches that helped them directly. The authors of these texts could potentially show greater awareness or sensitivity for adaptive survival.

**Table 1:** Specified survivor strategies identified in classical texts analyzed in this study.

Author	Title of Text	Survivors State What Helped them in Captivity
E. Ginzburg Gulag	Into the Whirlwind	“Poetry, they couldn’t take that away from me!… I would survive even this dungeon,” p. 220–221 [49].
A. Solzhenitsyn Gulag	Gulag Archipelago, I	“But those fellow prisoners… and that something that beats between your heart and theirs… and the birth within you,” p. 180–181 [50].
P. Levi Auschwitz	Survival in Auschwitz	Levi and Alberto formed a strong bond, all “organized” materials are equally divided, p. 125 They make a “manaschka” to transport soup, p. 132 [46].
C. Delbo Auschwitz	Auschwitz and After	Warming hands—each placed her hands under the arms of the one ahead “forming a single circulatory system,” p. 58 [47].
V. Frankl Auschwitz	Man’s Search for Meaning	“… any man can, even under such circumstances, decide what shall become of him–mentally and spiritually. He can retain his human dignity even in a concentration camp,” p. 87 [48].
R. Shumaker Hanoi POW	PBS, This Emotional Life	“I learned tools… it stood me in good stead for the rest of my life.” Televised quote [51].
J. Stockdale Hanoi POW	Thoughts of a Philosophical fighter Pilot	I knew that obligations, particularly love and self-sacrifice, were the glue that made a man whole in this primitive element,” p. 59 [52].

Of the classical survivor texts noted above, we chose three as the most suitable: the autobiography by Ginzburg [49], by Levi [46], and by Delbo [47]. A televised interview with Shumaker [51] was also included to diversify the source media. The starting points of thematic analysis were the specified strategies of these authors. These and other themes emerged during inductive analysis of the texts that resulted in three categories of themes: 1) Engagement included themes of interest, curiosity, appreciation, and noticing beauty; 2) Social relatedness covered empathy, compassion, helping, friendship, and love; and 3) Self-growth included internal and self-related growth. For consistent scoring of themes across texts, the themes are defined and illustrated with examples in the summary of Table 2.

**Table 2:** Categories, themes, and definitions in a thematic analysis applied to classical source texts used in this study.

Categories	Themes	Definitions and Examples of Themes
Engagement	Interest	Attention, stopping to notice; novelty; change in
		routine or context
	Curiosity	Exploration, seeking information
	Appreciation	Noticing positive qualities, benefits, of personal or cultural value
	Noticing beauty	Attraction, identification with, supporting life
Social Relatedness	Empathy	Sharing the same feelings as someone else
	Compassion	Wanting to help, wanting to alleviate suffering
	Helping	Statements of helping, offering and engaging in helping, recognizing need for help and helping
	Friendship	Positive relationship over time
	Love	Direct statements of love, identification with someone, risking one’s life for someone, saving someone’s life, strong bond over time,
Self-Growth	Internal	Spiritual, emotional, cognitive-knowledge; requires time
	Sense of self	Becomes enlarged beyond immediate self, takes others into account; requires time

### Scale development

#### Adaptive regulation and unpredictability in the chronic pain body environment

The development of regulation strategy items drew on several content areas: (1) qualitative review of adaptive survivor strategies in extreme environments of the classical survival literature that uncovered characteristic categories of engagement (specific strategies of interest, curiosity, appreciation, noticing beauty), social relatedness (strategies of empathy, compassion, helping, friendship, love), and self-growth [26]; (2) insight from the RISE Life Regulation intervention to train adaptive regulation strategies of engagement, social relatedness for self-growth and designing a good life [26]; (3) resources of existing scales that assess constituents making up engagement, social relatedness, and self-growth. This literature presents an extensive array of validated tests and experimental procedures on the specific components of engagement (interest, curiosity, etc) and social relatedness (empathy, compassion, etc). These do not address pain. (4) Research on unpredictability of pain and its attention demanding disruptive effects covered unpredictability content. The intervention team, expert in applying biobehavioral life regulation in clinical trials of chronic pain, undertook to review the relevant assessment literature and to develop the domains and items for the BLR scale. These are summarized in Table 3.

**Table 3:** Established non-pain scales and concepts consulted for the meaning of relevant concepts identified for engagement, social relatedness, self-growth, and pain unpredictability.

DOMAINS	SCALES & CONCEPTS–Relevant to Regulation Domains
REGULATION	
**Engagement**	
Interest	Increases with uncertainty, for information seeking [61]; complexity and instability increases interest [62]
Curiosity	Curiosity scale- factors for exploration, stress tolerance, empathy [63]; curiosity as motivational force [64]
Appreciation	Delayed appreciation of beauty-making connections [65]
Noticing Beauty	Effects on unity and agency of self [66]
**Social Relatedness**	
Empathy	Dynamic Empathy Questionnaire [67, 68]
Compassion	Self-Compassion Scale [10, 69]
Helping	Adult Prosocialness Scale [70]
Friendship	Sociality, community, & meaning making [71]
Love	Adult Attachment Scale for attachment security [72]
**Self-Growth**	
Eudaimonia	Hedonic and Eudaimonic Motives for Activities [73]; eudaimonic wellbeing [74]
Self-growth & Trauma	Posttraumatic Growth Scale [75]
UNPREDICTABILITY	EXPERIMENTAL & CONCEPTUAL Approach
**Pain Unpredictability**	Pain interruption & interference- experimental analysis [76]; Attention demanding [77]

It is interesting to note that the components of engagement (interest, curiosity, etc) and of social relatedness (empathy, compassion, etc) have no goals and express a future orientation to new information. This openness to experience and making connections appears to be particularly adaptive in unpredictable threatening environments as this way of being reinforces agency and supports self-growth. The construction of the BLR scale sought to develop a valid and reliable scale that could quantify and provide values for biobehavior life regulation in clinical samples living in the context of chronic pain and that could indicate the extent to which biobehavior regulation can be modified by interventions and ameliorative methods. A final review of items and their suitability in capturing the regulation strategies and pain unpredictability resulted in the 52 items that cover the three regulation domains of engagement, social relatedness, and self-growth as well as the unpredictability domain. These are listed in Table 4.

**Table 4:** Items covering the adaptive strategies of engagement, social relatedness, and self-growth in the presence of pain and the unpredictability of the body pain experience.

**Regulation Categories and Their Strategies**
**Engagement**
1. I continued to be interested in many things even when I was in pain.
2. I explored many new things and activities even when I was in pain.
3. Pain was my only interest when I was in pain.
4. Pain stifled my curiosity and desire to explore.
5. I appreciated the richness of life around me even in the presence of pain.
6. I sought out beauty even in the presence of pain.
7. Everything looked ugly when I was in pain.
8. I was involved in learning new things even when I was in pain.
9. I was blind to anything beautiful when I was in pain.
10. I sought out adventures even when I was in pain.
11. It was impossible to learn anything new when I was in pain.
12. I felt gratitude for things in my life even when I was in pain.
**Social Relatedness**
1. I had empathy even when I was in pain.
2. I had compassion for the suffering of others even when I was in pain.
3. I cared less about the suffering of others when I was in pain.
4. I was helpful to others even when I was in pain.
5. I sought out friends even in the presence of pain.
6. I helped others less when I was in pain.
7. I sought out people even when I was in pain.
8. I tried very hard to meet my obligations to others even when in pain.
9. I withdrew from people when I was in pain.
10. I was able to cherish and love others even when I was in pain.
11. I had compassion for my own suffering even when I was in pain.
12. It was more difficult to enjoy the company of others when I was in pain.
**Self-Growth**
1. Pain helped me reach for the best in me.
2. I transformed a setback into personal growth.
3. Pain made me feel inferior, whenever I had setbacks.
4. I sought to learn new skills to improve myself.
5. I sought to pursue excellence in what I did.
6. I believed that I had all the self-improvement skills I needed.
7. I had more empathy and compassion for others because of my challenges.
8. Pain helped me reach beyond my limitations and grow as a person.
9. I stuck with my goals despite my limitations.
10. I did not see excellence as a worthwhile goal for self-improvement.
11. Because of my pain, I did not have as many resources to help me improve as a person.
12. I looked for opportunities that could help me grow as a person.
**Unpredictability of Pain**
1. My pain started at any time of the day or night.
2. I could not tell how long my pain would last.
3. I could not depend on warning signals of coming pain.
4. My pain signals were very reliable; I could depend on them.
5. Negative experiences made my pain unpredictable.
6. I could not tell when a pain flare-up would come.
7. I could not tell how long I could stand the pain.
8. I could predict the comings and goings of my pain.
9. Pain made my ability to complete tasks unpredictable.
10. My relationship with my family was unstable, up-and-down because of pain.
11. Pain made my future goals and plans unpredictable.
12. I could predict all the activities my pain would let me do.
13. When I was in pain, my promises and commitments were unreliable.
14. I became careless about my self-care needs when I was in pain.
15. I could plan my activities for tomorrow, no matter how bad the pain was today.
16. Pain made my life unpredictable.

*Answer choices ranged from 1 = never, 2 = seldom, 3 = occasionally/sometimes/on occasion, 4 = often, 5 = always.

*Reliability and validity of the scale*. Convergent validity for the scale was evaluated by examining the correlation of total scores with related well-established constructs. A number of measures were chosen to assess convergent construct validity with the BLR subdomains of engagement, social relatedness, and self-growth as well as the unpredictability of pain. The scales selected for construct validation included:

- Chronic Pain Acceptance Questionnaire [78]
- Positive Relations with Others [79]
- Hedonic and Eudaimonic Motivation of Activities (HEMA) [73]
- Personal Growth [79]
- Intolerance of Uncertainty [80]
- Pain Catastrophizing Scale [81]

The Chronic Pain Acceptance Questionnaire (CPAQ-8) consists of a total of 8 items including an activity factor (4 items; Cronbach’s Alpha α = .88 within the current sample) that assesses functioning not restricted by pain and a pain willingness factor (4 items; Cronbach’s Alpha α = .60 within the current sample) that assesses willingness to have pain without controlling it. Ratings range from 0 (never true) to 6 (always true), with higher rating indicating greater activity engagement and pain willingness. The two subscales had acceptable or good internal consistency.

Positive Relations with Others and Personal Growth are two short seven-item subscales (14 items total) of the Psychological Well-Being Scale (PWS) by Ryff [82, 83]. Positive Relations with Others assesses warm, satisfying and trusting relationships that incorporate empathy, affection, and understanding. Items are rated on a range from 1 (strongly disagree) to 6 (strongly agree) with higher scores indicating a person has warm, satisfying and trusting relationships with others. Personal Growth assesses feelings of continued development, of growing, being open to experiences, and seeking to realize one’s own potential improvement of self. The rating scale ranges from 1 (strongly disagree) to 6 (strongly agree) with a higher scores indicating a person has a feeling of continued development and a sense of growing and realizing one’s potential. The PWS scales were tested with a national representative sample and have acceptable-to-good internal consistency. Within the current sample, Cronbach’s Alpha α = .81 for the Positive Relations with Others subscale and Cronbach’s Alpha α = .79 for Personal Growth.

Hedonic and Eudaimonic Motivation of Activities (HEMA) [73] was included because of its two-factor structure. Each subscale was analyzed separately. The hedonia factor (5 items) taps the pursuit of feeling good and relaxation that covers pleasure, enjoyment, and comfort. It functions as short-term emotional self-regulation and to restore normal affect. Eudaimonia (4 items) seeks to develop the best within oneself, personal growth, and aims for excellence and growth. Eudaimonia uplifts people beyond their usual boundaries. The hedonia and eudaimonia subscales each have acceptable-to-good psychometric properties (within the current sample, α = .77 and α = .87, respectively). Scale values range from 1 (not at all) to 7 (very much), with higher rating indicating greater pursuit of pleasure or comfort and relaxation for the hedonia factor and greater pursuit of excellence and seeking to develop the best in oneself for the eudaimonia factor.

The 12-item Intolerance of Uncertainty Scale (IUS-12) [80] taps anxiety related to the future and uncertainty that inhibits action in two factors of prospective and inhibitory anxiety. Prospective Anxiety taps an anxious component of IUS-12 such as “Unforeseen events upset me greatly (p. 113).” Inhibitory Anxiety covers an avoidance component of IUS-12 such as in “When it’s time to act, uncertainty paralyses me (p. 113).” This twelve-item scale ranges from 1 (Not at all characteristic of me) to 5 (Entirely characteristic of me) and has good psychometric properties (α. = 84 within the current sample), with higher scores indicating a greater intolerance of uncertainty.

The Pain Catastrophizing Scale (PCS) [81] consists of three components: rumination, magnification, and helplessness and has a total of 13 items. Rumination taps worries and attention to pain related thoughts. Magnification exaggerates the threat value of pain, such as “I think of other painful experiences (p. 526).” Helplessness covers inability to cope with pain. This thirteen-item scale with range values of 0 to 4 (higher scores indicate greater pain catastrophizing) has excellent psychometric properties (α = .92 within the current sample).

For convergent construct validation, the meaning of the constructs of the BLR subdomains of engagement, social relatedness and self-growth appear to resemble the meaning of the constructs for CPAQ-8, Positive Relations with Others, Personal Growth and HEMA. The unpredictability of pain construct appears to resemble the meaning of the construct for ambiguity in the IUS-12 and PCS. The extent of convergence between BLR subdomains and construct validation scales noted here will be demonstrated in subsequent correlational analyses. Scales to assess divergent construct validity were not included.

### Procedures

A total of 202 participants completed the BLR questionnaire and relevant validation scales. The participants were drawn from two populations: patients enrolled in the Phoenix Veterans Affairs Health Care System (PVAHCS), (n = 112) and two private clinics in Phoenix that included a pain clinic (Sample 2a; n = 40) offering interdisciplinary pain treatment and a general mental health clinic (Sample 2b; n = 50). The two community clinic samples were combined into a single sample (n = 90) to provide an independent non-VA sample for the confirmatory factor analysis (CFA). The community samples did not differ from each other on any demographic variables. The PVAHCS sample was more diverse than the combined community sample in terms of race, Chi square = 12.56, *p* < 0.05, and reported longer duration of pain, *t*(184) = -2.69, *p* < 0.01. Participants from the combined community sample had fewer years of education than the PVAHCS sample, *t*(152) = -2.93, *p* < 0.01.

Study procedures, participant recruitment, and consenting were approved by the Institutional Review Board (IRB) of the PVAHCS for Veteran participants. The IRB of Arizona State University approved the conduct of this study for the community clinics. Recruitment of Veteran participants occurred through provider referrals, announcements, and approved fliers posted at designated places at the PVAHCS Chronic Pain Wellness Center, and at the Carl T. Hayden Medical Center. Referrals at the private clinics came from intake personnel.

All study participants who were self- or physician-identified as having chronic pain, meeting minimal chronic pain criteria, and were within the appropriate age range were accepted for consenting. See Table 5 for specific inclusion/exclusion criteria. Following consenting, prospective participants were screened for exclusion criteria that included physical and mental health limitations as well as current psychological therapies that could bias participant responses. Chart diagnoses of chronic pain were reviewed but were not required for participant inclusion. Only after prospective participants had passed the suicidality, alcohol dependence, and psychosis screens, and met all inclusion criteria, were they enrolled for participation in the study.

**Table 5:** Inclusion and exclusion criteria for RBP participants.

Inclusion	Exclusion
Age 18–75, Self or physician identified as having chronic pain	1. Active suicidality (Columbia Suicide Severity Rating Scale–C-SSRS) [84]
	2. Active alcohol (scoring guidelines of AUDIT-C) [85]
	3. Active psychosis (Psychosis Screener) [86]
	4. Current severe disabling illness (recent or impending surgery, acute illness
	5. Neurocognitive conditions (e.g. dementia, Parkinson’s, stroke)
	6. Concurrent therapies: exposure, cognitive behavior, acceptance.

### Data analytic plan

#### Factor analysis

Exploratory Factor Analysis (EFA) was employed in SPSS version 29 to examine the initial factor structure [87–89]. To assess the sampling adequacy for factor analysis, item correlations, the Kaiser-Meyer-Olkin (KMO), Bartlett’s test of sphericity, and communalities (> .60) were assessed [90, 91]. Specifically, item level correlations were evaluated for their magnitude (r’s > .30) to assess the presence of sufficient linear relationships [92]. To evaluate sampling adequacy, KMO values greater than .70 and a significant Bartlett’s test of sphericity were used [93, 94].

Additionally, a scree plot and the K1 rule were used to best determine the number of factors appropriate to retain [95]. Specifically, the scree plot was used to determine the number of factors above the point of inflexion [96]. The K1 rule was used to examine factors with eigenvalues greater than 1.00 for retention [97].

An oblique (Oblimin) rotation was used to allow for factor correlation. Item loadings of .32 or above were considered significant, whereas loadings of .50 or above were considered strong and significant [98, 99]. Items were eliminated if they demonstrated no significant loading or had significant cross loadings >.30.

To confirm the factor structures of the scale determined by the EFA, confirmatory factor analysis (CFA) was employed in Mplus 8.1 in the original sample and a secondary sample using FIML estimation with Robust Errors to accommodate any minor deviations from normality. Model fit was evaluated using multiple indices. Excellent model fit was defined as CFI and TLI values close to .95 or above, RMSEA values close to .06 or below, and SRMR values close to .08 or below [100]. Adequate model fit was defined as CFI and TLI values close to .90 or above, and RMSEA values close to .10 or below.

## Results

Descriptive statistics on the characteristics of each sample are reported in Table 6. Items were reviewed by members of the study team (JGS and LC) who identified that the reverse coded items were difficult to comprehend and complicated the scoring. Reverse coded items have also been shown to statistically impact outcomes [101], therefore these items were eliminated. Five items were also removed due to redundant content. A total of 32 items remained for the initial exploratory factor analysis (EFA), as shown in Table 7.

**Table 6:** Sample characteristics.

	Sample 1 (*n* = 112)	Sample 2 (n = 90)	Sample 2a (*n* = 40)	Sample 2b (*n* = 50)
Age	54.5 (12.3)	52.26 (13.9)	45.49 (13.8)	57.48 (11.8)
%Male	78	-	-	-
% African American Race	10.4	-	-	-
% Asian Race	.9	-	-	-
% Native American Race	.9	2.6	3.1	2.2
% White Race	80.2	94.8	96.9	93.3
% Other Race*	7.5	2.6	-	4.4
% Hispanic Ethnicity	8.2	14.9	11.1	19.4
Education (years)	14.72 (1.7)	13.68 (2.3)	13.24 (2.3)	14.09 (2.2)
Pain duration (years)	20.37 (13.3)	15.48 (11.42)	14.97 (9.41)	15.89 (12.9)
% Pain: Constant	61.7	50	45.9	53.2
% Pain: Intermittent	6.5	3.6	2.7	4.3
% Pain: Both Constant and Intermittent	31.8	46.4	51.4	42.6

*Note*. Sample 1 = patients receiving treatment at the Phoenix Veterans Affairs Health Care System (PVAHCS); Sample 2 = Combined community sample made of up Sample 2a and Sample 2b. Sample 2a = Community pain clinic; Sample 2b = Community mental health clinic. Information on participant gender was not collected in the community clinic samples.

*Other was an option provided to participants to select

**Table 7:** Exploratory factor analysis in VA sample from 32 items (n = 112).

	Factor 1	Factor 2
**I continued to be interested in many things even when I was in pain.**	.69	-.03
**I explored many new things and activities even when I was in pain.**	.82	-.03
**I appreciated the richness of life around me even in the presence of pain.**	.82	.18
**I sought out beauty even in the presence of pain.**	.78	.24
I was involved in learning new things even when I was in pain.	.72	.23
I sought out adventures even when I was in pain.	.68	.22
I felt gratitude for things in my life even when I was in pain	.79	.28
**I had empathy even when I was in pain.**	.71	.18
I had compassion for the suffering of others even when I was in pain.	.69	.20
**I was helpful to others even when I was in pain.**	.68	-.06
I sought out friends even in the presence of pain.	.76	-.09
**I sought out people even when I was in pain.**	.78	.01
**I tried very hard to meet my obligations to others even when in pain.**	.62	.06
**I was able to cherish and love others even when I was in pain.**	.62	.24
I had compassion for my own suffering even when I was in pain.	.47	.21
Pain helped me reach for the best in me.	.62	.15
**I sought to learn new skills to improve myself.**	.62	.12
When in pain, I sought to pursue excellence in what I did.	.72	.10
I had more empathy and compassion for others because of my pain	.69	.16
**Pain helped me reach beyond my limitations and grow as a person.**	.73	.04
**I stuck with my goals despite my limitations.**	.77	-.03
**I looked for opportunities that could help me grow as a person.**	.76	.05
**My pain started at any time of the day or night**	-.02	.34
**Negative experiences made my pain unpredictable**	-.26	.53
My pain flare-ups were unreliable.	-.28	.42
The comings and goings of my pain were unpredictable	-.13	.40
**Pain made my ability to complete tasks unpredictable.**	-.35	.55
**My relationship with my family was unstable, up-and-down because of pain.**	-.40	.48
Pain made my future goals and plans unpredictable.	-.45	.48
**When I was in pain, my promises and commitments were unreliable.**	-.60	.43
I became careless about my self-care needs when I was in pain.	-.62	.30
**Pain made my life unpredictable.**	-.47	.66

*Note*. Shaded items were dropped as they did not meet criteria for retention; bolded items (19 items) were retained for final confirmatory analysis after team review.

### Exploratory factor analysis

Exploratory Factor Analysis (EFA) evaluated the extent to which the factor structure was consistent with the four hypothesized factors (engagement, social relatedness, self-growth, and unpredictability) using the initial 32 items. Results showed the KMO index (.889) fell within the adequate range of shared variance [90] and Bartlett’s test of sphericity was significant (*df* = 496, *χ*^2^ = 2512.86, *p* < .001), confirming adequacy of sample size. Evaluation of the communalities showed most items were greater than .60, confirming adequate covariance to employ factor analysis. Nearly all items showed significant correlations (all *p*’s < .001) and sufficient covariation among items (*r*’s > .30), meeting the assumption of a linear relationship between variables. A scree plot was used to help determine the number of factors to retain. The scree plot suggested that two factors should be retained, although all solutions through six-factors showed eigenvalues over 1.00.

Results of the exploratory factor analysis with two, three, and four-factor solutions were analyzed in the VA Sample (n = 112) using Oblimin rotation. Items with factor loadings < .32, or cross-loadings ≥.30 were excluded from the scale, resulting in 30 total items retained. The four-factor solution had eigenvalues ranging from 12.77 to 1.03 and accounted for 55.94% of the total variance. The three-factor solution had eigenvalues ranging from 12.76–1.22 and accounted for 52.24% of the total variance. The two-factor solution had eigenvalues of 12.72 and 2.72 and accounted for 48.23% of the total variance. The two-factor solution was preferred because the results of the scree plot indicated a leveling off after 2 factors, and the solutions with 3 and 4 factors had weak loadings, did not correspond to the subscales originally hypothesized in the *a priori* item development, and were difficult to interpret. see Table 7.

The EFA was repeated on the retained 30 items using the VA Sample 1 (*n* = 112). In the two-factor solution, which explained 48.96% of the overall variance, Factor 1: Pain Regulation, accounted for 40.37% of the variance, and Factor 2: Pain Unpredictability, accounted for 8.59% of the variance. However, the 30 retained items appeared prohibitive for implementation and widespread use. Therefore, the study team again reduced items that seemed redundant, resulting in a scale with 19 items, a listed in Table 8.

**Table 8:** Confirmatory factor analysis in VA and community samples.

	Sample 1 VA (n = 112)		Sample 2 Community (n = 90)	
Factor 1: Pain Regulation				
I continued to be interested in many things even when I was in pain.				
I explored many new things and activities even when I was in pain.	.82			.68
I appreciated the richness of life around me even in the presence of pain.	.80			.80
I sought out beauty even in the presence of pain.				
I had empathy even when I was in pain.	.70			.59
I was helpful to others even when I was in pain.	.67			.59
I sought out people even when I was in pain.	.79			.78
I tried very hard to meet my obligations to others even when in pain.				
I was able to cherish and love others even when I was in pain.	.59			.53
I sought to learn new skills to improve myself.				
Pain helped me reach beyond my limitations and grow as a person.	.74			.51
I stuck with my goals despite my limitations.	.74			.61
I looked for opportunities that could help me grow as a person.				
Factor 2: Pain Unpredictability				
My pain started at any time of the day or night	.29			.40
Negative experiences made my pain unpredictable	.48			.42
Pain made my ability to complete tasks unpredictable.	.67			.80
My relationship with my family was unstable, up-and-down, because of pain.	.67			.63
When I was in pain, my promises and commitments were unreliable.	.74			.74
Pain made my life unpredictable.	.76			.75

*Note*. Shaded items were dropped based on high item-level correlations suggesting content redundancy. The final scale included 14 items: 8 items for Pain Regulation and 6 items for Pain Unpredictability.

### Confirmatory factor analysis

Although not originally the hypothesized solution, the 19-item scale was evaluated using Confirmatory Factor Analysis (CFA) in MPlus 8.1 for a 2-factor solution based on the EFA results. The initial CFA results in the VA sample (*n* = 112) showed high correlations among a few of the items in Factor 1, indicating additional redundancy in item content and resulting in reduced model fit per the modification indices. With the exclusion of five items from Factor 1, the 2-factor solution provided adequate fit to the data (χ^2^[76] = 137.45, *p* < .001, CFI = .91, TLI = .90, RMSEA = .085, SRMR = .07). The two factors were negatively correlated (*r* = -.59, *p* < .001), suggesting that the subscales measure distinct, though related, constructs. See Table 6. Cronbach’s alpha was used to examine the internal consistency of responses across the revised subscales: .90 for Pain Regulation (8 items); and .78 for Pain Unpredictability (6 items).

The 2-factor CFA using the retained 14 items was repeated in Sample 2 from the community clinics (*n* = 90). The 2-factor solution provided similarly adequate fit to the data (χ^2^[76] = 113.86, *p* < .01, CFI = .91, TLI = .89, RMSEA = .07, SRMR = .08). The two factors were negatively correlated (*r* = -.45, *p* < .001), suggesting that the subscales measure distinct, though related, constructs. See Table 8. Cronbach’s alpha was used to examine the internal consistency of responses across the revised subscales: .84 for Pain Regulation (8 items); and .78 for Pain Unpredictability (6 items).

### Reliability and validity of the scale

In both samples, factor scores were computed in SPSS for construct validity analyses. Pearson correlations were computed between the BLR Scale subscales, and measures of theoretically-related constructs (Table 9).

**Table 9:** Correlations between the BLR scale and theoretically-related measures in VA and community samples.

	BLR VA Sample			BLR Community Sample	
	n = 112			n = 90	
Measure	Pain Regulation	Pain Unpredictability	Pain Regulation		Pain Unpredictability
Intolerance of Uncertainty Scale	-.45**	.45**	-.24*		.35**
Happiness (Hedonia) subscale (HEMA)	.32**	-.19*	.28*		-.05
Eudaimonia (Growth) subscale (HEMA)	.54**	-.32**	.34**		-.01
Pain Catastrophizing scale	-.51**	.65**	-.39*		.45**
Chronic Pain Acceptance- Active engagement	.64**	-.43**	.59**		-.31**
Chronic Pain Acceptance- Pain Willingness	-.09	.26*	-.02		.32**
Personal relationships (RYFF)	.49*	-.38**	.49**		-.28**
Personal growth (RYFF)	.59**	-.43**	.45**		-.15

Note

*p < 0.05

**p < 0.01.

The Pain Regulation subscale had moderate positive correlations with HEMA happiness (*r* = .32, *p <* 0.001), HEMA Eudaimonia (*r* = .54, *p <* 0.001), pain acceptance-active engagement (*r* = .64, *p <* 0.001), higher levels of personal growth (*r* = .59, *p <* 0.001), and positive relationships (*r* = .49, *p <* 0.001), suggesting that those who endorse employing and utilizing strategies to engage are helpful in the context of pain and increase facets of well-being. The Pain Regulation subscale also had moderate negative correlations with the Intolerance of Uncertainty Scale (IUS-12, r = -.45, *p <* .001), and the Pain Catastrophizing Scale (r = -.51, *p <* .001), suggesting that those with higher scores on this factor tend to engage less in maladaptive thought patterns specific to their pain.

The Pain Unpredictability subscale had moderate positive correlations with scale scores on the IUS-12 (r = .45, *p <* .001) and the PCS (r = .65, *p <* .001). Pain Unpredictability had negative correlations with pain acceptance-active engagement (r = -.43, *p <* .001), personal growth (r = -.43, *p <* .001), and rewarding personal relationships (r = -.38, *p <* .001). Conceptually, this suggests that unpredictability of chronic pain is associated with more negative thoughts, behaviors, and social isolation while also being distinct from anxiety about the future and pain catastrophizing.

The correlations and patterns were relatively consistent in both the VA and community samples, although there were a few notable differences. In the community sample, pain unpredictability was not correlated with the HEMA subscales or with personal growth.

## Discussion

The BLR scale was developed to address a gap in existing pain assessment scales by shifting the focus of assessment from symptoms and limitations to a person’s inner adaptive regulatory capacities within the aversive unpredictable internal environment of chronic pain. Three content sources were consulted for the development of the items for the BLR scale: (1) survivor accounts of adaptive survival in extreme environments (2) the RISE Life Regulation intervention for chronic pain [26], and (3) existing scales covering the themes of Engagement, Social Relatedness, and Self-Growth as described in Table 3. The themes of adaptive functioning as identified in qualitative analysis of survival in extreme environments and as applied in the RISE intervention and existing non-pain thematic relevant scales itemized in Table 3 yielded the themes of Engagement, Social Relatedness and Self-Growth. These core themes along with the unpredictability of the body environment of pain, formed the *a priori* subdomains for the BLR scale. This study presents the initial validation of the BLR scale using Exploratory and Confirmatory Factor Analyses.

The most interesting finding in the EFA is that the three features of adaptive extreme experiences formed a single Pain Regulation factor, Factor 1. The specific content of engagement, social relatedness, and self-growth did not form sub-factors but tapped a feature that all three had in common; participants responded similarly in the context of pain. The 8 items in Factor 1 represent the combined themes of adaptive regulatory strategies of reaching beyond the self in engagement, social relatedness, and self-growth. All three content domains express an inclination and approach beyond the self. They express a future orientation that is prospective rather than reactive, is open to new information, possibilities, and direction to explore with a sense of agency. Engagement items express approach through interest, exploration, appreciation and noticing beauty. Social relatedness is similarly future oriented and open to connecting with people, as seen in empathy, helping, seeking out people, and to cherish and love. Self-growth is seen in new learning and creativity that reach beyond limitations. This Pain Regulation factor expresses agency and simultaneously reaches beyond the self. Thus, regulation taps what will enlarge engagement in all three spheres, depending on individual inclinations and opportunities in the environment.

Another common feature of Factor 1 is that all items form a gradient along the dimension of orientation/engagement that is not dictated by the experience of pain. It is a prospective anticipation gradient that is open ended for what may fit idiosyncratic interest, sociality and self-growth and for opportunities the environment might offer.

Factor 1 meets the biological definition of the living organism as self-maintaining and that supports the universal properties of living organisms, the most important being autonomy and regulation of autonomy, self-restoration, and self-preservation. Factor 1, Pain Regulation, supports autonomy and self-maintenance in its prospective orientation for opportunities that support the organism’s wellbeing.

An extensive existing literature addresses the concepts and scales that assess the components of engagement, social relatedness, and self-growth, as shown in Table 3. Not only do scales for interest and curiosity exist, studies have shown how they are related to unpredictability [62] and information seeking [61] Our search for social relatedness identified empathy scales [67, 68], the prosocialness scale [70], meaning making [71], and how all of these relate to environmental demands. Most noteworthy, these scales demonstrated the relevance of Factor 1 items as active participants in regulation activities provoked by features of context. Moreover, various studies also showed how scale values were related to the activation of the default network (DN) and its sub-networks. Thus, the following examples illustrate the relationships between scale values and the DN, such as for curiosity and the DN [9], appreciation [65], compassion [10], helping [102], creativity [11], and noticing beauty [103]. These findings point to the DN as a potential life regulation cortical network. It will be recalled that the DN is activated by spontaneous self-guided thought that is decoupled from attention-focused tasks and salience evoking stimuli that also characterize pain. This decoupling fosters self-generated thinking and such powerful tools as representation, memory, planning, and social cognition, thereby expanding the scope of life beyond pain.

The two factors of the BLR assessed what they were intended to assess. Construct validity analysis showed that the Pain Regulation subscale correlated positively with happiness and eudaimonic self-growth, with personal growth, with pain acceptance, and with positive relations with others. This subscale endorses self-growth, pain acceptance-active engagement, and positive relations as strategies for well-being in the context of pain. At the same time, participants scoring high on the Pain Regulation subscale were less inclined to engage in maladaptive thinking as seen in the negative correlations with intolerance of uncertainty and pain catastrophizing.

Construct validation of the Pain Unpredictability subscale showed moderate positive correlations with intolerance of uncertainty and with pain catastrophizing, thus tapping a maladaptive dimension of pain related distress of the PCS and unpleasant anxiety in the IUS. While Factor 1 of the BLR represents anticipatory life regulation, Factor 2, Pain Unpredictability, taps the inability to anticipate and make predictions about the future. Pain Unpredictability is conceptually distinct from both pain catastrophizing and intolerance of uncertainty. Pain catastrophizing and intolerance of uncertainty involve maladaptive reactions to pain [104] and uncertainty [105] respectively. Pain Unpredictability assesses an inability to anticipate the future, tapping the loss of a future orientation.

While the Unpredictability subscale remained fairly consistent across the VA and community samples, one difference arose with the community sample in which pain unpredictability was not correlated with the HEMA subscales or with personal growth. The difference may be due to differing durations of pain in the samples. The PVAHCS sample reported longer duration of pain and thus the PVAHCS had more time to develop a self-growth approach to chronic pain.

This study had a number of important limitations including small sample size; limited diversity of study population in ethnicity, race, and education; a lack of identification of specific pain conditions; and a lack of analysis of sensitivity to change for diverse treatment interventions. Another limitation of the study is that, once the items of the scale were defined, the scale was not pre-tested in a small sample prior to performing the factor analyses. For the scale to find wider utility it needs to be tested with a larger sample size with diversity in race, ethnicity, education, and socioeconomic status and with identification of specific pain conditions. Its utility should be demonstrated in diverse practice settings ranging from academic pain centers to community primary care clinics. This study has made a first attempt to address diversity at these various levels. Future research is needed to determine the sensitivity of the BLR scale to detect change for a variety of therapeutic interventions including those that aim to reduce symptoms as well as interventions that aim to enhance innate life regulation capacities. In addition, future research is needed to elucidate how the BLR scale may be able to guide treatment planning. This study has demonstrated that unpredictability is a salient factor in chronic pain and that adaptive regulation is an identifiable life orientation that can be engaged and expanded. The BLR scale itself can help identify regulation resources of engagement, social relatedness, and self-growth as well as vulnerability in the unpredictability of pain. While this study has provided the initial validation for each of the two identified factors, additional research is needed to identify the clinical utility of each of the BLR factors in assessing progress towards treatment goals and predicting response to treatment. The two factors may also be further explored and refined in future qualitative research that captures individuals’ use of anticipation skills in the context of pain and the quality and degree of their experience of unanticipated/unpredictable pain. Given the unique domains assessed by the BLR scale, researchers may consider use alongside standard scales that assess symptoms, reactions to pain, or psychological flexibility, however, additional research as described above is needed to determine which combinations and scenarios will be most fruitful.

What began as a hypothesis about adaptive strategies in extreme human-made environments and comparable features in chronic pain led to the development of the BLR scale to assess the presence of adaptive strategies and regulation in chronic pain. The scale expands assessment capabilities to cover the “insider” experiences of being well in the presence of chronic pain, and elucidates the involved regulation resources. The development of the BLR scale is, thus, a first attempt to fill a gap in assessment approaches. The BLR scale may help to illuminate the nature of biobehavioral regulation in the experience of chronic pain and the unpredictability of chronic pain. This scale may advance pain treatment and assessment by providing a tool for assessing the methods with which patients subjectively regulate pain and the subjective magnitude of unpredictability of chronic pain.
